# Cluster analysis identifies three urodynamic patterns in patients with orthotopic neobladder reconstruction

**DOI:** 10.1371/journal.pone.0185255

**Published:** 2017-10-18

**Authors:** Kwang Hyun Kim, Hyun Suk Yoon, Wan Song, Hee Jung Choo, Hana Yoon, Woo Sik Chung, Bong Suk Sim, Dong Hyeon Lee

**Affiliations:** 1 Department of Urology, Ewha Womans University College of Medicine, Seoul, Korea; 2 Department of Urology, Ewha Womans University, Mokdong Hospital, Seoul, Korea; Ludwig-Maximilians-Universitat Munchen, GERMANY

## Abstract

**Purpose:**

To classify patients with orthotopic neobladder based on urodynamic parameters using cluster analysis and to characterize the voiding function of each group.

**Materials and methods:**

From January 2012 to November 2015, 142 patients with bladder cancer underwent radical cystectomy and Studer neobladder reconstruction at our institute. Of the 142 patients, 103 with complete urodynamic data and information on urinary functional outcomes were included in this study. K-means clustering was performed with urodynamic parameters which included maximal cystometric capacity, residual volume, maximal flow rate, compliance, and detrusor pressure at maximum flow rate. Three groups emerged by cluster analysis. Urodynamic parameters and urinary function outcomes were compared between three groups.

**Results:**

Group 1 (n = 44) had ideal urodynamic parameters with a mean maximal bladder capacity of 513.3 ml and mean residual urine volume of 33.1 ml. Group 2 (n = 42) was characterized by small bladder capacity with low compliance. Patients in group 2 had higher rates of daytime incontinence and nighttime incontinence than patients in group 1. Group 3 (n = 17) was characterized by large residual urine volume with high compliance. When we examined gender differences in urodynamics and functional outcomes, residual urine volume and the rate of daytime incontinence were only marginally significant. However, females were significantly more likely to belong to group 2 or 3 (P = 0.003). In multivariate analysis to identify factors associated with group 1 which has the most ideal urodynamic pattern, age (OR 0.95, P = 0.017) and male gender (OR 7.57, P = 0.003) were identified as significant factors.

**Conclusions:**

While patients with ileal neobladder present with various voiding symptoms, three urodynamic patterns were identified by cluster analysis. Approximately half of patients had ideal urodynamic parameters. The other two groups were characterized by large residual urine and small capacity bladder with low compliance. Young age and male gender appear to have a favorable impact on urodynamic and voiding outcomes in patients undergoing orthotopic neobladder reconstruction.

## Introduction

Orthotopic neobladder reconstruction is now the preferred option for urinary diversion after radical cystectomy for bladder cancer [1]. Despite the complexity in surgical procedure and postoperative management, patients with orthotopic neobladder void via native urethra and showed better quality of life compared to patients with incontinent urinary diversion [2]. Orthotopic neobladder provides low pressure reservoir with adequate capacity preserving urinary continence and upper urinary tract function. Several studies have demonstrated excellent long-term results with stabilized reservoir capacity and satisfactory continence rates [3–5]. However, assessment of functional outcomes in patients with orthotopic neobladder is a complex task and the method has not been standardized. For reliable and structured evaluation, previous studies have developed and used bladder cancer- and neobladder-specific questionnaires [6–8]. These questionnaires usually focus on functional outcomes such as urinary incontinence or quality of life after surgery.

Urodynamics provide objective information on lower urinary tract function by the measurement of various volume and pressure parameters. Urodynamic evaluation in patients with orthotopic neobladder is not a new effort and multiple studies have been conducted to investigate long-term changes in urodynamic parameters or to compare differences in urodynamic parameters between orthotopic neobladders with various intestinal segments [9–11]. Meanwhile, most urodynamic studies have described the orthotopic neobladder as an excellent urinary reservoir with adequate volume and low pressure [12–15]. However, orthotopic neobladders are created out of intestinal segments and show innate differences from original bladder in terms of sensory and motor functions. In fact, individuals with orthotopic neobladder have diverse urodynamic and voiding features.

While previous studies have described the urodynamic parameters of orthotopic neobladders, we have limited information on the characterization of urodynamic or voiding patterns in patients with orthotopic neobladder. In this study, we aimed to classify patients with orthotopic neobladder according to urodynamic parameters using cluster analysis and investigated urinary functional outcomes in each group.

## Materials and methods

A retrospective review of medical record and analysis was performed after obtaining Ewha Womans University Mokdong Hospital institutional review board (2015-10-010-001).

### Patient inclusion

From January 2012 to November 2015, 142 patients with bladder cancer underwent radical cystectomy and orthotopic neobladder reconstruction by a single surgeon (DHL). Informed consent was waived due to retrospective design and data were analyzed anonymously after removal of patient identifiers. All patients underwent radical cystectomy with a standard open surgical approach and the extent of lymphadenectomy included the obturator fossa and the external, internal and common iliac vessels up to the ureteric crossing. Nerve sparing was attempted when it did not compromise cancer control. The vaginal sparing procedure was performed in all female patients. A Studer pouch was used for orthotopic substitution [16]. Briefly, an approximately 60 cm segment of the ileum was isolated and the proximal 15 cm was used for the afferent limb. Ureteroileal anastomosis was performed using the Nesbit technique. For neobladder reconstruction, the distal part was detubularized and double folded to construct a U-shaped pouch. From November 2013, we performed video urodynamic testing postoperatively, as part of a comprehensive functional evaluation of ileal neobladder. Usually, the test was performed at 3–6 months after surgery when most patients achieved daytime continence. However, the test was deferred or unperformed depending on medical condition or consent of patients. Of a total of 142 patients, 103 with complete urodynamic data and information on voiding function were included in this study.

### Video urodynamic study and evaluation of voiding function

All urodynamic tests were conducted by a single specialized nurse (HJC) according to the standards of the International Continence Society [17]. Multichannel urodynamic system (MMS Solar, Medical Measurement System, Enschede, The Netherlands) was used for the urodynamic test. Voiding diary and continence status were evaluated at the time of urodynamic testing. Functional bladder capacity was used as a reference for urodynamic testing. Continence was classified into daytime and nighttime and defined as the use of zero pads. The pressure-flow test was performed with an air-charged 7-Fr dual lumen urethral catheter and a 7.5-Fr water-filled rectal PVC catheter with an introducer. The patients were placed in a semi-sitting position and the bladder was filled at a filling rate of 50 ml/min with normal saline of room temperature. Using maximal functional bladder capacity on voiding diary as reference, maximal bladder capacity was measured at a feeling of fullness of the bladder or urine flowing through urethra. Compliance was calculated by dividing volume change by detrusor pressure change. In cases of sudden increase or fluctuation of detrusor pressure, the detrusor pressure was measured after pressure curve was stabilized. The patient was asked to void with comfortable position for each individual. Maximum flow rate and residual urine volume were measured. We also measured maximum abdominal pressure during the voiding phase as most patients with ileal neobladder use abdominal straining to empty their bladder. On video urodynamics, the incidence and characteristics of vesicoureteral reflux (VUR) were evaluated. Along with VUR, the incidence of febrile urinary tract infection (UTI) was also evaluated. Febrile UTI was defined as a positive urine culture with at least 10^4^ colony-forming units per milliliter of urine and an elevated body temperature (≥38°C).

### Statistical analysis

Cluster analysis was performed using R version 3.2.5 (http://www.r-project.org). K-means, unsupervised learning method, was used for clustering of urodynamic parameters which included maximal cystometric capacity, residual volume, maximal flow rate, compliance, and detrusor pressure at maximum flow rate. The NbClust package [18] was used to determine the optimal number of clusters. NbClust provides 30 indices for deciding the right number of clusters. In our analysis, NbClust computed 26 multiple tests; both 2 and 3 were proposed as the best number of clusters by 8 and 7 indices, respectively, with highest frequencies (S1 Fig). We determined the optimal number of clusters to be 3 after analysis with various clusters. Clinical variables, urodynamic parameters, and voiding status between the 3 clusters were compared. Qualitative variables were compared using Chi squared test and quantitative variables were compared using one-way ANOVA. Multivariate logistic regression analysis was used to identify clinical factors predicting ideal urodynamic patterns after surgery. For these analyses, the Statistical Package for Social Science for Windows, version 18.0 (SPSS, Inc., Chicago, IL, USA) was used and a P-value <0.05 was considered significant. All P-values were two-sided.

## Results

**Fig 1:**
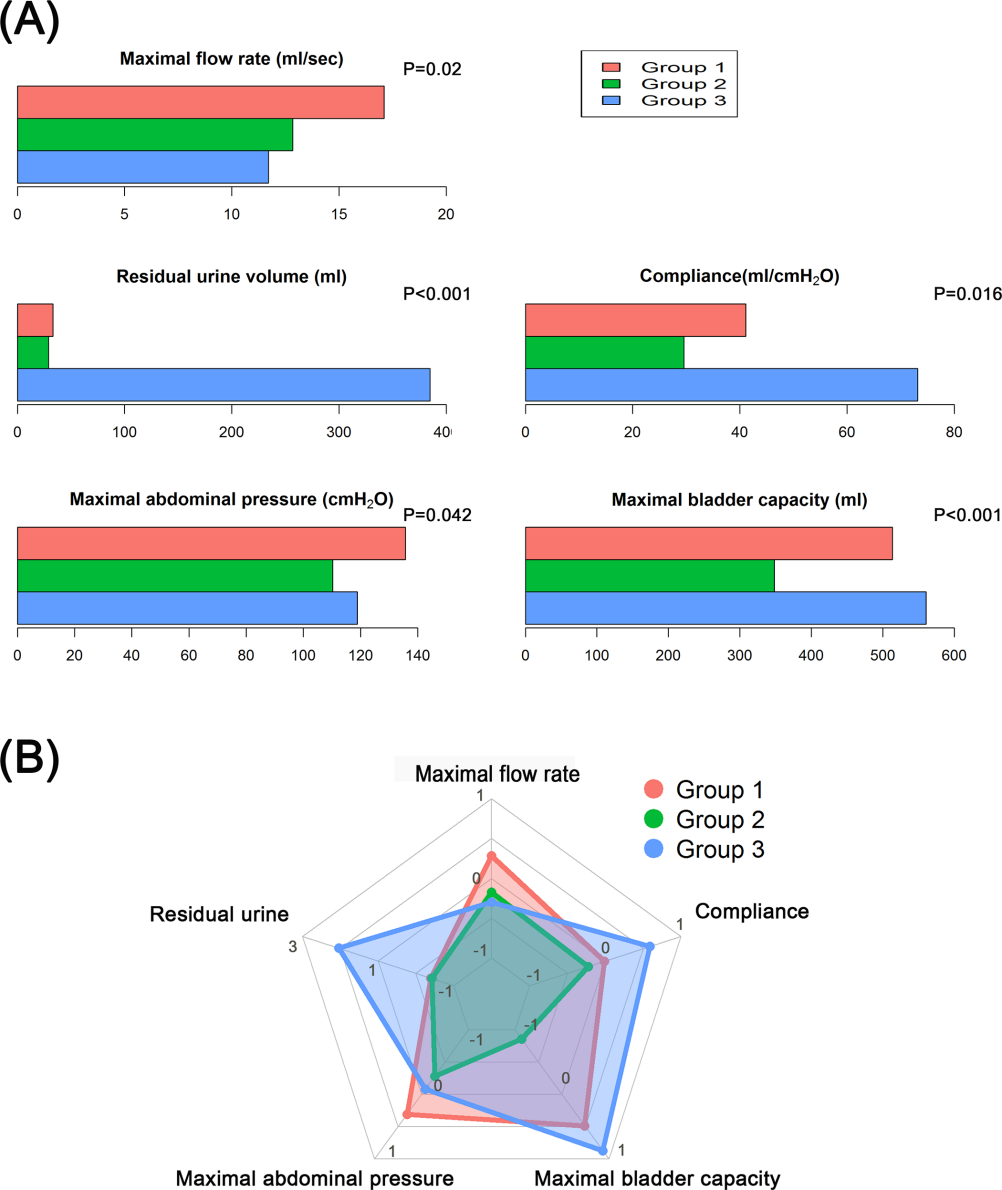
(A) Comparison of urodynamic parameters between the 3 groups. One-way ANOVA was used for statistical analysis. (B) Radar plot of urodynamic parameters among the 3 groups using standardized z-score.

Median age at surgery was 64 (interquartile range, 56–71) and 80 (77.7%) were male. Median time from surgery to urodynamic testing was 5 months (interquartile range, 3–10). Among all patients in this study, mean maximal bladder capacity, residual urine volume, and compliance were 453.7 ml, 89.5 ml and 41.6 ml/cmH_2_O, respectively. Three groups emerged by cluster analysis using 5 urodynamic parameters, which were significantly different between the 3 groups (Fig 1). Group 1 (n = 44) had most ideal urodynamic pattern among the 3 groups. In group 1, mean maximal bladder capacity and residual urine volume were 513.3 ml and 33.1 ml, respectively. Patients in this group also showed the highest maximum flow rate at 17.0 ml/sec. Group 2 (n = 42) was characterized by small bladder capacity with low compliance. Patients in group 2 had the smallest mean maximal bladder capacity (348.1 ml) and the lowest mean bladder compliance (29.5 ml/cmH_2_O). They had the higher incidence of incontinence during both daytime (31.0%) and night time (83.3%) than patients in group 1 (Table 1). The last group (n = 17) had the largest mean residual urine volume of 384.6 ml. They also had the largest mean maximal bladder capacity (560.4 ml) and the highest bladder compliance (73.1 ml/cmH_2_O). Of the 17 patients in group 3, 6 (35.3%) required routine clean intermittent catheterization.

**Table 1:** Clinical and urinary functional outcomes of the 3 groups.

	Group 1 (n = 44)	Group 2 (n = 42)	Group 3 (n = 17)	P value
Age (year), mean ± SD	59.6±11.4	64.6±10.6	64.9±7.2	0.055
Gender, n (%)				0.003
male	41 (93.2)	29 (69.0)	10 (58.8)	
female	3 (6.8)	13 (31.0)	7 (41.2)	
Postoperative period (month)				0.628
median, IQR	6, 3–13	4, 3–10	5, 3–8.5	
BMI (kg/m2), mean ± SD	24.1±2.9	23.8±2.8	25.5±3.3	0.139
Hypertension, n (%)	16 (36.4)	18 (42.9)	8 (47.1)	0.702
Diabetes n (%)	7 (15.9)	7 (16.7)	3 (17.6)	0.986
Daytime incontinence, n (%)	5 (11.4)	13 (31.0)	7 (41.2)	0.022
Nightime incontinence, n (%)	29 (65.9)	35 (83.3)	15 (88.2)	0.076
Vesicoureteral reflux, n (%)	12 (27.3)	23 (54.8)	5 (29.4)	0.022
Febrile UTI, n (%)	11 (25.0)	11 (26.2)	8 (47.1)	0.203

SD = standard deviation, BMI = body mass index, UTI = urinary tract infection

On video urodynamic testing, VUR was found in 40 patients (38.8%). VUR was bilateral in 22 patients (55.5%, 22/40). Of 62 renal units with VUR, 23 (37.1%) were of grade III or higher. The incidence of VUR was highest (54.8%) among patients in group 2, which was characterized of small bladder capacity and low compliance. However, the incidence of febrile UTI was highest (47.1%) in group 3, which was the group that showed the largest residual urine. Also, the incidence of febrile UTI was not significantly different according to the presence of VUR (25.4% vs. 35.0% in VUR (-) vs. VUR (+), P = 0.374) (Table 1). Urodynamic variables were also not different between patients with and without febrile UTI (S1 Table).

Patients in group 1 had the most ideal urodynamic parameters and presented with the lowest incidence of incontinence. Among the forty-four (44/103, 42.7%) patients in group 1, 93.2% were male. When we examined gender differences in urodynamics and functional outcomes, residual urine volume and the rate of daytime incontinence were only marginally significant (Table 2). However, females were significantly more likely to belong to group 2 or 3 (P = 0.003). Age, body mass index, comorbidities such as hypertension or diabetes, and postoperative time did not differ between the three groups (Table 1). When we performed multivariate logistic regression analysis to identify factors associated with group 1, age (OR 0.95, P = 0.017) and male gender (OR 7.57, P = 0.003) were identified as significant factors (Table 3).

**Table 2:** Gender differences in urodynamic parameters and urinary functional outcomes.

	Male (n = 80)	Female (n = 23)	P value
Urodyamic parameters, mean ± SD			
Maximal bladder capacity (ml)	462.3±110.7	423.9±155.4	0.186
Residual urine volume (ml)	74.8±130.6	140.3±180.9	0.116
Maximal flow rate (ml/sec)	14.3±8.8	15.0±11.0	0.738
Compliance (ml/cmH_2_O)	38.3±42.7	53.3±80.5	0.237
Maximal abdominal pressure (cmH_2_O)	126.5±44.0	108.7±36.1	0.080
Urinary functional outcomes, n (%)			
Daytime incontinence	64 (80.0)	14 (60.9)	0.059
Nighttime incontinence	16 (20.0)	9 (39.1)	0.841

SD = standard deviation

**Table 3:** Logistic regression analysis to predict factors associated with being assigned to group 1, the most ideal urodynamic pattern.

	Univariate		Multivariate	
	Odd ratio	P value	Odd ratio	P value
Age	0.95 (0.91–0.99)	0.02	0.95 (0.91–0.99)	0.017
Gender				
Female	Ref		Ref	
Male	7.00 (1.92–25.46)	0.003	7.57 (2.03–28.19)	0.003
Hypertension	0.72 (0.32–1.61)	0.432		
Diabetes	0.92 (0.32–2.66)	0.888		
Body mass index	0.98 (0.86–1.11)	0.769		
Postoperative period (month)	1.01 (0.98–1.03)	0.394		

## Discussion

Cluster analysis is an unsupervised machine learning method that partitions a dataset into groups of individuals or objectives which are similar to each other. Determining the optimal number of groups is a critical step in cluster analysis and a wide variety of methods exit. In our study, NbClust was used to select the optimal number of clusters in the dataset [18]. Analysis revealed the best number of groups was 2, but using two groups only identified patients with normal voiding and large residual urine volume. We performed further analysis with various numbers of clusters and determined that 3 was the optimal number of groups. A cluster number of 3 was the second most commonly proposed number by NbCluster and the three groups had significantly different patterns of urodynamic parameters.

Cluster analysis with 3 groups identified an additional group of patients characterized by small bladder capacity and low compliance that is similar to overactive bladder. The urodynamic features of this group were somewhat contrary to known features of orthotopic neobladder. It might be due to the relatively short postoperative period time in our cohort. However, short postoperative period time could not fully explain the voiding feature of all patients in this group, which accounted for approximately 40% of the entire cohort. Previous studies have described the presence of neobladder over-activity and the association with nocturnal incontinence and urge incontinence [19,20]. A recent study has shown the efficacy of intravesical botulinum toxin injection in the treatment of patients with neobladder over-activity and urinary incontinence [21]. In our urodynamic evaluation, we could not definitively define neobladder over-activity due to various forms of intraluminal pressure change and urodynamic artifact. However, we found that patients in this group had significantly smaller capacity and lower compliance than patients with group 1 who were thought to have the most ideal urodynamic parameters. We speculate that clinical factors or intestinal length, diameter, or elasticity could contribute to this urodynamic character resulting in higher rates of urinary incontinence. On the other hand, urinary incontinence itself might be a cause of small capacity by providing insufficient expanding capacity in orthotopic neobladder.

In our study, the nighttime incontinence rate was approximately 75% and considerably higher than that of previous reports, which were mostly within 30% [3–5]. However, differences in the definition used to define incontinence or the method used for data acquisition, such as telephone interviews, in previous studies may have contributed to such differences. Evaluation of functional outcomes using validated questionnaires found that only 17.7% of patients with ileal neobladder were fully continent [22]. Kretschmer et al [23] also found that continence rates were lower than previously described when objective continence definitions were used; in their study, nocturnal continence rate was 36.3%. A recent study which used a questionnaire specific to neobladder reported that 78.4% of patients used a pad at nighttime, which is comparable to our results [8]. These results suggest that nighttime incontinence is one of the most common side effects after orthotopic neobladder reconstruction. Nighttime incontinence adversely affects quality of life of patients with orthotopic neobladder and remains a significant problem [24]. It has been known that the absence of detrusor-sphincter reflex is a main cause of nighttime incontinence following surgery. Various efforts have been made to improve nighttime incontinence. Results from a small, randomized study has suggested the beneficial effects of oxybutynin and verapamil [25]. Low doses of oral desmopressin have also been found to be effective in reducing the number of nocturnal voids and nighttime incontinence [26]. We summarized the results of previous studies which treated the dysfunctional voiding of patients who underwent orthotopic neobladder reconstruction (Table 4).

**Table 4:** Summary of previous studies which investigated the treatment of dysfunctional voiding in patients with orthotopic neobladder.

	Dysfunctional voiding	No. patients	Intervention	Result
Ghoneim et al [27]	Nocturnal incontinence	32	imipramine hydrochloride 25mg	improved in 8 patients (25%)
El-Bahnasawy et al [25]	Nocturnal incontinence	20	oxybutinine	improved in 14 patients (70%)
El-Bahnasawy et al [25]	Nocturnal incontinence	20	verapamil	improved in 11 patients (55%)
Goldberg et al [26]	Nocturnal incontinence	31	desmopression 0.1mg	mean number of night time awakening decreased from 2.5/night to 1.5/night
Hoag et al [21]	Overactive ileal neobladder	4	intravesical onabotulinumtoxinA	varying degrees of subjective improvements

With improved understanding of female pelvic and urethral anatomy, orthotopic neobladder became the diversion of choice for women after radical cystectomy. It has been known that approximately 65% of women undergoing radical cystectomy can be a candidate for orthotopic neobladder reconstruction [28]. In selected female patients, the long-term oncologic safety of orthotopic neobladder and satisfactory functional outcomes have been shown [29]. However, urinary retention is still a major functional problem in women with orthotopic neobladder [30]. In our results, 30.4% of female patients were in group 3, which was characterized by large residual urine volume, while 12.5% of male patients were assigned to group 3. Furthermore, more than half of female patients were in the group with small capacity and low compliance, and only three females (13.0%) belonged to the ideal voiding group. Although these results are unsatisfactory compared to previous reports on female orthotopic neobladder [29,31], a recent large center study also identified significant prevalence of voiding dysfunction in women with orthotopic neobladder [32]. In our analysis, it appears that increasing age and female gender negatively affect urodynamic and functional outcomes in patients with orthotopic neobladder. Increasing age has been associated with voiding dysfunction in multiple studies [8,32]. In terms of gender, we could not find significant gender differences in urodynamic and voiding feature (Table 2). However, cluster analysis and multivariate logistic regression analysis suggested that urinary functional outcomes of female patients undergoing orthotopic neobladder reconstruction is inferior to that of male patients.

This study is not devoid of limitations. First, while the study cohort which included more than 100 patients is relatively large compared to previous studies of urodynamics in patients with orthotopic neobladder [9,11–13], the size of our cohort may be small for the cluster analysis. With more patients with urodynamic results, we could have identified a greater number of clusters with specific voiding characteristics. Second, due to its retrospective nature, the postoperative time interval of the urodynamic test was not consistent and relatively short. Considering that urodynamic profiles improve during the first year after surgery [3,33], urodynamic tests at an early postoperative time may not reflect the matured functional state of orthotopic neobladder. We commonly performed urodynamic tests at 3–6 months after surgery as comprehensive urodynamic evaluation and for early intervention of voiding dysfunction. Urodynamic tests at 1 year after surgery would best represent the matured and stabilized state of orthotopic neobladder. However, most patients become accustomed to the new voiding environment at 3–6 months and it has been reported that urodynamic results were not significantly different within 6–12 months after surgery [34]. Our results are valuable given that we identified various features of voiding pattern in patients with orthotopic neobladder, regardless of time intervals. Third, structured and validated questionnaires were not used for the evaluation of voiding function. However, the specialized urologic nurse who performed urodynamic tests consistently interviewed patients and recorded their voiding status. Although the severity of urinary incontinence was not analyzed, we strictly defined the continence state as zero pad use. Fourth, while our study was focused on classifying and describing voiding patterns based on urodynamic findings, we did not investigate the treatments or training for improving voiding function in patients with orthotopic neobladder. However, we found that elderly or female patients were more likely to have unfavorable voiding patterns after neobladder reconstruction. This information would be of great value for physicians who counsel patients undergoing radical cystectomy and urinary diversion. Finally, the clustering of the 3 groups was not externally validated. If clustering was validated in other cohorts of patients with orthotopic neobladder, it would be possible to investigate the characteristics of each group in greater depth. Ultimately, a clear classification system can lead to further research to improve functional outcomes or quality of life for each group.

In conclusion, cluster analysis identified three groups with different urodynamic and voiding characteristics. While approximately half of patients had an ideal urodynamic pattern with adequate capacity and compliance, the other two groups were characterized by large residual urine and small capacity bladder with low compliance. Clinical factors such as young age and male gender were associated with an ideal urodynamic pattern. We recommend that physician counsel patients with this information.

## Supporting information

S1 Fig Optimal number of clusters proposed by NbClust, which calculated 26 indices. Among indices, 8 and 7 proposed 2 and 3, respectively, as the optimal number of clusters.
(TIF)

S1 Table Urodynamic variables and patterns according to the presence of febrile urinary tract infection. (DOCX)
