# Supplementary figures and images for: Cluster analysis identifies three urodynamic patterns in patients with orthotopic neobladder reconstruction
Source: PLoS One. 2017 Oct 18;12(10):e0185255. doi: 10.1371/journal.pone.0185255 (PMC5646783; doi:10.1371/journal.pone.0185255)

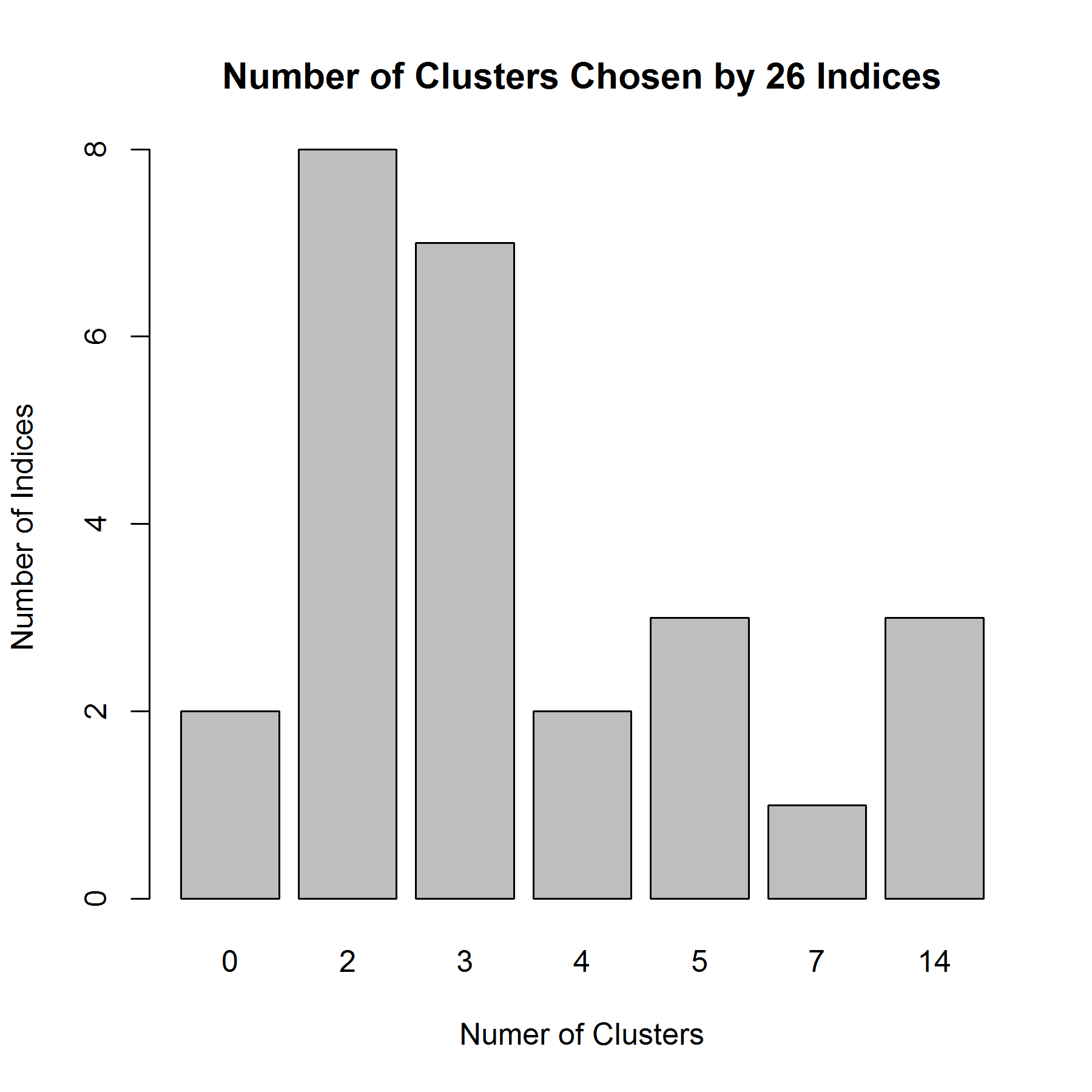

Supplement: S1 Fig — Among indices, 8 and 7 proposed 2 and 3, respectively, as the optimal number of clusters. (TIF) [file pone.0185255.s001.tif]
