# Supplementary material for: Cluster analysis identifies three urodynamic patterns in patients with orthotopic neobladder reconstruction
Source: PLoS One. 2017 Oct 18;12(10):e0185255. doi: 10.1371/journal.pone.0185255 (PMC5646783; doi:10.1371/journal.pone.0185255)
Supplement: S1 Table — (DOCX) [file pone.0185255.s002.docx]

Table S1 – Urodynamic variables and patterns according to the presence of febrile urinary tract infection

|  | Febrile UTI (+) | Febrile UTI (-) | P Value |
| --- | --- | --- | --- |
| Maximal capacity (mL) | 473.3±136.8 | 445.6±115.8 | 0.300 |
| Maximal abdominal pressure (cmH2O) | 131.4±45.0 | 118.8±41.8 | 0.178 |
| Maximal flow rate (mL/sec) | 13.8±9.6 | 14.7±9.2 | 0.178 |
| Residual urine (cc) | 118.7±172.4 | 77.4±131.5 | 0.245 |
| Compliance (ml/cmH2O) | 47.4±72.9 | 39.3±43.3 | 0.486 |
| VUR, n (%) |  |  | 0.296 |
| absent | 16 (53.3) | 47 (64.4) |  |
| present | 14 (46.7) | 26 (35.6) |  |
| Cluster, n (%) |  |  | 0.203 |
| group 1 | 11 (36.7) | 33 (45.2) |  |
| group 2 | 11 (36.7) | 31 (42.5) |  |
| group 3 | 8 (26.7) | 9 (12.3) |  |
